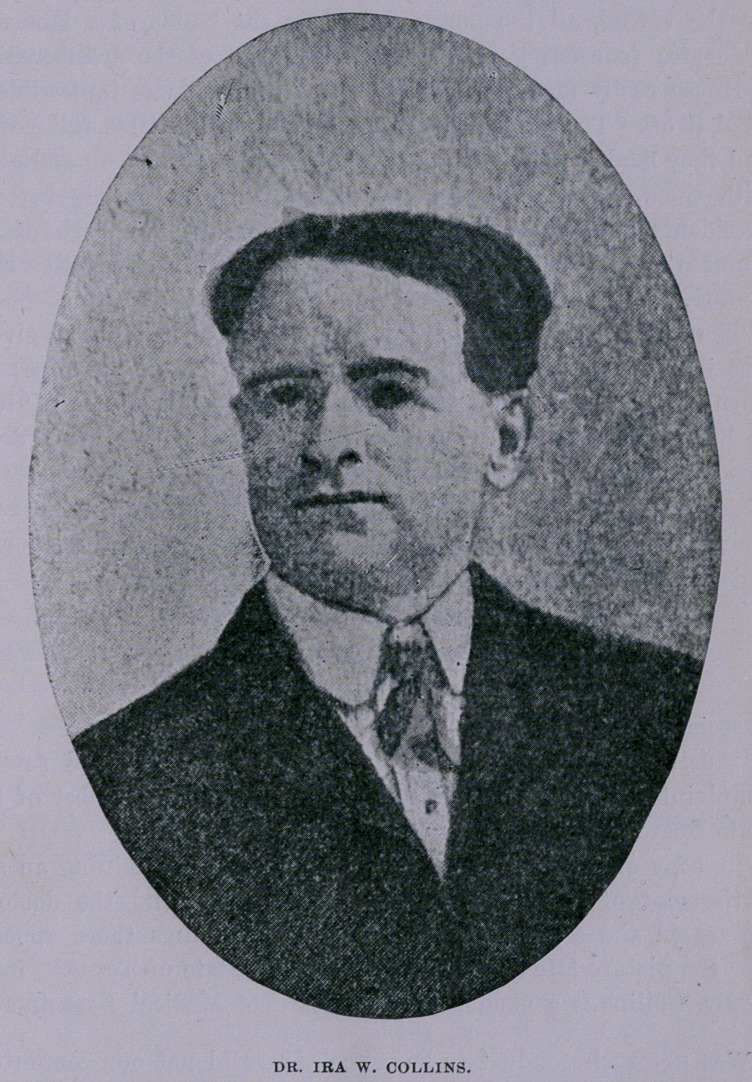# First Fruits of “Unification”

**Published:** 1907-11

**Authors:** 


### EDITORIAL DEPARTMENT.

### FIRST FRUITS OF "UNIFICATION."

### The Degradation of Medicine the Shame of Texas.

*Oh, the Pity of It!*

At the urgent and most persistent solicitation of the Committee on Legislation, and after a battle of many days duration, the Thirtieth Legislature passed the now notorious One Board Bill, a bill to regulate the practice of medicine and *suppress quackery.*

This committee consisted of the President of the Texas State Medical Association (ex-officio Chairman) ; the Secretary of the State Association and editor of the Association's *Journal* (also ex-officio) ; Dr. Cantrell, now President of the Association, and two other gentlemen.

While the bill was in the Legislature a number of zealous members, thoroughly in sympathy with the "new dispensation" idea, and the proselyting propaganda for "unifying the profession," leveling all distinctions in medicine,—a movement originating with the Octopus, launched from Chicago, and pushed by Walking Delegate McCormack, attended the sessions and lobbied manfully for the bill,—a bill recognizing all the pathies, even the Osteos.

The committee invited the irregulars to join them in the effort to get a bill which they felt cocksure would *suppress quackery.*

Well, they got it. They just would have it!

The Board is composed of five members of the State Association, two Homeos, two Eclectics, one Physio-Med., and one Osteopath.

It was feared that the Governor would veto the bill, and he was besieged by the legislative committee and their associates, and urged to sign it. He was assured that "everybody wanted it," so he told me, whereas the facts have developed that nobody wanted it except those who were instrumental in getting it. The mass of members of our State Association, through the House of Delegates, at Fort Worth, in April, 1906, repudiated it; the representatives of the Homeopathic "School" (Association) repudiated it; and the Osteopaths, I am informed, regret throwing away their opportunity of getting a separate Board.

Well, we have the Board, a crazy-quilt, Joseph's coat mosaic, a *pot pourri* of fish, flesh and fowl and a sprinkling of red herring, and behold the consequences! Read this: We are suppressing quackery, all right.

(Advertisement.)

### WHAT IS THE MATTER WITH YOU?

DR. IRA W. COLLINS, PHYSICIAN IN CHIEF A. T. STILL INFIRMARY, MEDICAL EXAMINER FOR THE STATE OF TEXAS.

From *The El Paso Herald*, October 12, 1907.

"Did you ever stop to think that the blood built up and made every part of you, and that if this blood stops circulating in any part of you because the nerves which force the blood to that part is congested at the spine, you have dead blood there, and you are sick. This nerve may have been overworked from taking some kind of drug, or some other poison into the system, or you may even have eaten too much, or taken some kind of a stimulant, such as pepper, tea, coffee or alcohol, and the nerve congested at the spine to rest itself, and the fluid of that joint became exhausted and dried and it could not restore itself. Now, there is no such thing as a remedy in the world. All that any drug can do to these nerves is to stimulate them, and exhaust what vitality they have or deaden them. Now, all you need to do is to have this nerve freed at the spine, and it will become as strong as it ever was, and as soon as this nerve can restore the circulation you have no more decaying blood, and you are well again. That is all there is to disease.

"Now, you must come soon enough and not wait until the spine has grown together and the organ entirely decayed, and you will surely get well, for it is being proven every day. You all know what happens to an egg when the hen is setting on it, and she leaves it too long; it begins to decay instead of chemically uniting and building up cell life. Now, it has not heat enough. Now, that is exactly what is taking place in your blood. It is decaying, and if this process takes place in the lungs, consumption results; in the eyes, you have various kinds of sore eyes; in the head, catarrh; in the throat, bronchial troubles; in the stomach, stomach trouble; in the kidneys, kidney trouble; in the liver, liver trouble; in the intestines, constipation and appendicitis; in glands around them, typhoid fever and other intestinal troubles; in muscles, rheumatism. When these congestions take place in the small of the back and hips, then this decaying blood causes various kinds of female troubles in ladies.

"Now, it is being proven every day that when an Osteopath frees these nerves, they force the circulation again, and build the patient up, and he is well again. But you must come soon enough

DR. IRA W. COLLINS.

for treatment, because if these organs have decayed until there is hardly anything left of them, you can never hope to be as strong again as you would have been, although the disease may still be arrested and cured. Now, do not put off coming until you have tried everything else, and it is entirely too late. GET BUSY. ATTEND TO IT RIGHT NOW."

It is no use to say "I told you so." The *Texas Medical Journal* and its editor, backed by the real friends of the Association, have opposed this sort of thing twenty years; fought it off, sometimes, single-handed, but always with success, notably in Fort Worth in 1896 (see Transactions). But under the reorganization, the plan originating with and launched by the Octopus, whereby all the voting, all the power, is put in the hands of a House of Delegates (consisting of about two per cent of the membership), a House easily manipulated, if not packed,—it was impossible to head it off. The House gave the legislative committee full power, and they made a deal with the irregulars and insisted on affiliating with them. Well, the Governor appointed the Board—two ex-presidents and three members of the Regulars, two each of the Homeos and Eclectics and one Physio and one Osteo,—all representative men.

The young Osteopath gentleman whose advertisement I give a free insertion, I am told, is fairly representative of his class; yet when he uses his appointment to head his advertisement, to "turn an honest penny," and preaches a new kind of pathology,—"dried blood" and "dead nerves," and "decaying organs," the cause of all diseases—even those that afflict the "ladies"—our folks raise their hands in horror and surprise, and beseech the Governor to make him "behave himself."

The following I clip from the *Galveston News*:

"COMPLAIN OF MEDICAL EXAMINER.

"Special to *The News*.

"Austin, Texas, Oct. 18.—The Governor has received several complaints relative to advertising matter used by a member of the State Board of Medical Examiners.

"A copy of the *El Paso Herald* was received containing an advertisement of Dr. Ira W. Collins, Osteopath, and the doctor's picture in the center of it. Among other things there appears over the picture 'Medical Examiner for the State of Texas.'

"Dr. Collins is a member of the Board of Medical Examiners."

Was not such conduct to be expected? Did not our committee know that it is the nature and custom of these fellows to advertise? They surely knew what they were going up against when they took the Osteos to their embrace. And even should the Governor remove this gentleman and appoint another, what guarantee is there that he will not do likewise?

But can the Governor remove him? I think not. There is nothing in the bill to give him power to do so. In advertising the "doctor" has violated no statute, nor is he amenable to the State Medical Association. The Osteos are not bound by our code, and the offender is out of reach of trial by court medical. The Governor must needs stand by his appointment, and the State Medical Association will just have to take its medicine. It is a case of Diomedeian necessity.

It was a most unfortunate mistake; misdirected zeal. I give my distinguished friends of the committee credit for honesty of purpose, but, being under the domination of the Octopus, run by an apostate Homeopath, they were simply misled. The pathies can never homologate with regular medicine. We have sacrificed pride, self-respect, all the noble traditions of our once proud profession and falsified its record for centuries for what? Medicine has been degraded, and its friends and the State of Texas shamed.

Really, I do not know whether "it is to laugh"—or to weep. The situation is as pitiful as it is ludicrous.

And this is the young gentleman who has been chosen to examine the graduates of our great University Medical College. He has been assigned to the chair of hygiene, which was a mistake. He should have been given pathology and physical diagnosis. By the by,—have the framers and friends of the bill ever reflected that it turns the drugless element loose, and removes all restrictions,—licensing those Osteopaths who pass examination to practice medicine in all its branches—with as well as without drugs, and that, too, without their ever having been examined in materia medica, therapeutics and the principles of practice? Verily, they have made a mess of "regulating the practice" and "suppressing quackery."

THE OCTOPUS AND OSTEOPATHY.—The Octopus conceived the unification idea, and launched the proselyting propaganda. Apostle McCormack preached it in the sanctum of regular medicine in every State, and the absorption of the irregulars by their advice,—including the Osteos, is now an accomplished fact; a kind of "benevolent assimilation." At the same time, the Octopus, to turn an honest penny, has published a book on its own account, showing the Osteopaths to be a good thing to let alone, and the *Journal A. M. A.* (the Octopus) is carrying this advertisement:

### "OSTEOPATHY.

"A JUDICIAL INQUIRY INTO ITS CLAIMS, OPINION OF JUDGE STERLING B. TONEY, OF KENTUCKY.

"This brochure declares the so-called 'osteopathic' method of healing to be nothing but a complete system of charlatanism, empiricism and quackery, calculated and designed to impose upon the credulous, superstitious and ignorant. Expert testimony shows that 'osteopathic treatment' as applied to many diseases is positively dangerous, inhuman and barbarous, and that such treatment, unless administered under the supervision and direction of a person learned and skilled in medicine, would be of no benefit to a patient, but, on the contrary, would do harm. This pamphlet proves that the whole 'secret' of osteopathy, in a nutshell, is manipulation—MASSAGE. To the physician who wishes to become familiar with the 'mysteries of osteopathy,' information derived from this pamphlet is invaluable.

"The decision here reported was subsequently reversed. This pamphlet is still circulated because of the information contained, which seems not to be obtainable elsewhere.

"Prices—Sent prepaid on receipt of 15c per copy, two for 25c; $6 per 100. Stamps accepted.

"American Medical Association Press, 103 Dearborn Ave., Chicago."

Oh, Consistency! Thy name is Octopus!

## Figures and Tables

**Figure f1:**